# Supplementary material for: Characterizing cell-type spatial relationships across length scales in spatially resolved omics data
Source: Nat Commun. 2025 Jan 3;16:350. doi: 10.1038/s41467-024-55700-1 (PMC11699133; doi:10.1038/s41467-024-55700-1)
Supplement: Supplementary file 3 — Supplementary Software 1 [file 41467_2024_55700_MOESM3_ESM.zip › CRAWDAD/plots/supplementary/S7_cluster_corr_heatmap_PKHL_vs_PBVN.pdf]

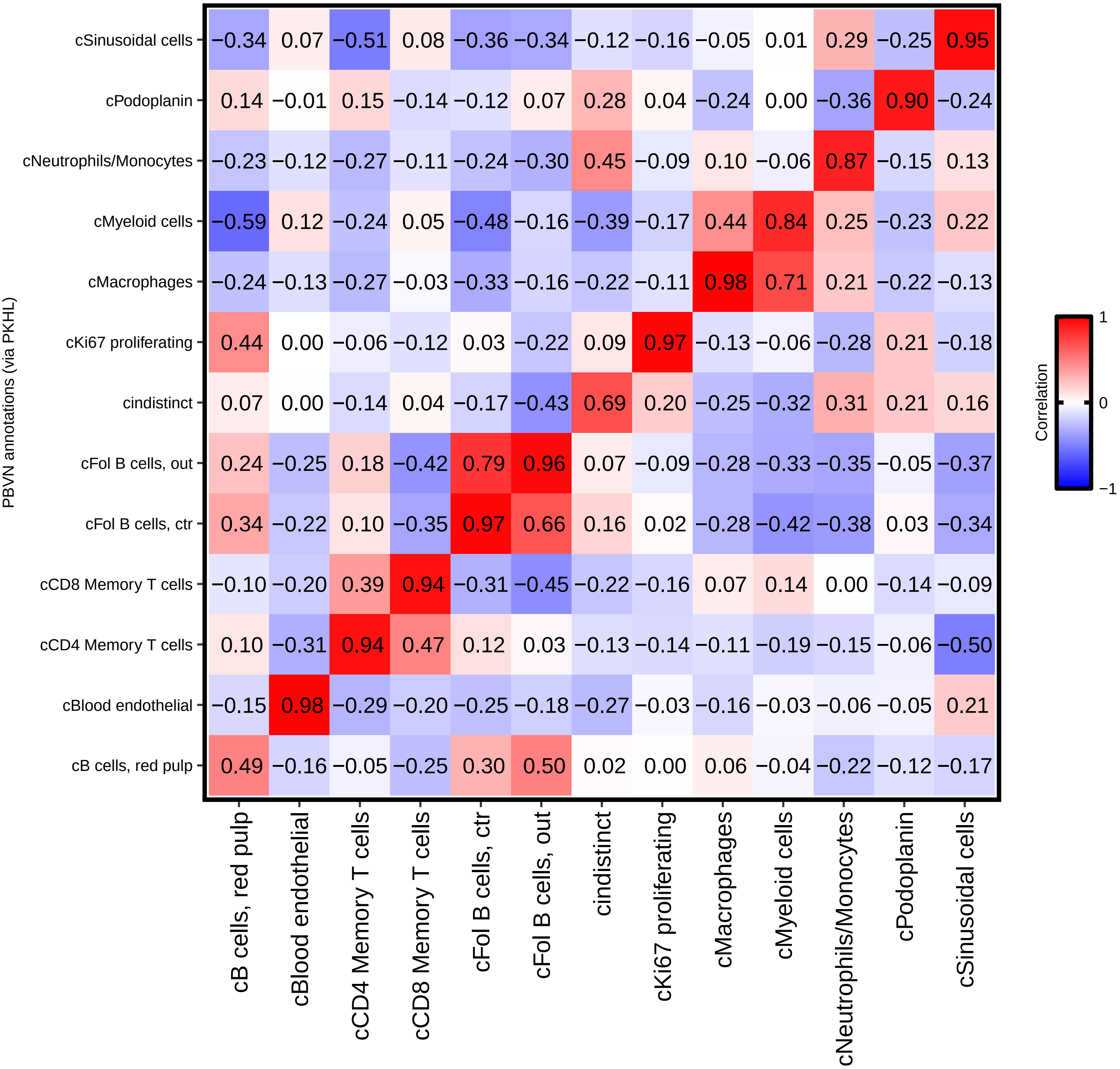

cSinusoidal cells
cPodoplanin
cNeutrophils/Monocytes
cMyeloid cells
cMacrophages
cKi67 proliferating
cindistinct
cFol B cells, out
cFol B cells, ctr
cCD8 Memory T cells
cCD4 Memory T cells
cBlood endothelial
cB cells, red pulp
PBVN annotations (via PKHL)
−0.34 0.07 −0.51 0.08 −0.36 −0.34 −0.12 −0.16 −0.05 0.01 0.29 −0.25 0.95
0.14 −0.01 0.15 −0.14 −0.12 0.07 0.28 0.04 −0.24 0.00 −0.36 0.90 −0.24
−0.23 −0.12 −0.27 −0.11 −0.24 −0.30 0.45 −0.09 0.10 −0.06 0.87 −0.15 0.13
−0.59 0.12 −0.24 0.05 −0.48 −0.16 −0.39 −0.17 0.44 0.84 0.25 −0.23 0.22
−0.24 −0.13 −0.27 −0.03 −0.33 −0.16 −0.22 −0.11 0.98 0.71 0.21 −0.22 −0.13
0.44 0.00 −0.06 −0.12 0.03 −0.22 0.09 0.97 −0.13 −0.06 −0.28 0.21 −0.18
0.07 0.00 −0.14 0.04 −0.17 −0.43 0.69 0.20 −0.25 −0.32 0.31 0.21 0.16
0.24 −0.25 0.18 −0.42 0.79 0.96 0.07 −0.09 −0.28 −0.33 −0.35 −0.05 −0.37
0.34 −0.22 0.10 −0.35 0.97 0.66 0.16 0.02 −0.28 −0.42 −0.38 0.03 −0.34
−0.10 −0.20 0.39 0.94 −0.31 −0.45 −0.22 −0.16 0.07 0.14 0.00 −0.14 −0.09
0.10 −0.31 0.94 0.47 0.12 0.03 −0.13 −0.14 −0.11 −0.19 −0.15 −0.06 −0.50
−0.15 0.98 −0.29 −0.20 −0.25 −0.18 −0.27 −0.03 −0.16 −0.03 −0.06 −0.05 0.21
0.49 −0.16 −0.05 −0.25 0.30 0.50 0.02 0.00 0.06 −0.04 −0.22 −0.12 −0.17
cB cells, red pulp
cBlood endothelial
cCD4 Memory T cells
cCD8 Memory T cells
cFol B cells, ctr
cFol B cells, out
cindistinct
cKi67 proliferating
cMacrophages
cMyeloid cells
cNeutrophils/Monocytes
cPodoplanin
cSinusoidal cells
PKHL
Correlation
1
0
−1
